# Diplomas in Radiography

**Published:** 1921-07-30

**Authors:** 


### THE EDITOR'S LETTER-BOX.

**Correspondence on all subjects is invited, but we cannot in any way be responsible for the opinions expressed.**

### Diplomas in Radiography.

*To the Editor of* The Hospital.

Sir,—I note in your paper an account of the first meeting of the Society of Radiographers. I was very much surprised to see that a certain number of people have already been awarded the diploma, as it has been impossible for myself, and other practising *x*-ray operators, to obtain any information about the Society's examination, though we made inquiries, long before the examination was held, as to when and where it was possible to qualify.

It would be interesting to know through what organs the Society advertises itself, as the required information was not in your paper. Do the newly qualified candidates hold the full diploma? and, if so, could not intending candidates see last year's questions, so as to have some rough idea of the type and extent of knowledge required? I suppose one may take it for granted that operators who have passed the Guy's Hospital examination in radiography and electrotherapy will be admitted automatically to the Register; their standard is, of course, well known (they being the pioneers), and this is the usual procedure with newly formed societies. I imagine the same would hold good for people with ten or more years' experience.

I remain, Sir, yours faithfully,

Spark-gap.

